# Human endogenous retroviruses antibody signatures in severe asthma phenotypes

**DOI:** 10.1007/s12026-026-09855-2

**Published:** 2026-10-08

**Authors:** Milena Fais, Rachele Campus, Elena Rita Simula, Maria Carmina Pau, Barbara Piras, Pietro Pirina, Leonardo A. Sechi

**Affiliations:** 1 Department of Biomedical Sciences, University of Sassari, Sassari, Italy; 2 Department of Medicine, Surgery and Pharmacy, University of Sassari, Sassari, Italy; 3 Microbiology and Virology unit, Azienda Ospedaliera Universitaria Sassari, Sassari, Italy

**Keywords:** Asthma, Severe asthma, HERV-K, HERV-W, HERV-H, Circulating antibodies, Antigenic peptides

## Abstract

Human endogenous retroviruses (HERVs) represent approximately 8% of the human genome and have been increasingly implicated in the pathophysiology of immune-related disorders. In this study, we investigated the humoral response against specific antigenic peptides derived from HERV-H, HERV-K, and HERV-W in the plasma of patients across different asthma phenotypes, including non-severe and severe cases, compared to healthy controls (HCs). Our results show that antibody levels against HERV-W were significantly low in the asthmatic population, showing a progressive downregulation that was more pronounced in severe cases. Conversely, a distinct and robust upregulation was uniquely observed for HERV-H in severe asthma cases, reaching a 100% positivity rate. Furthermore, HERV-K antibody levels were significantly lower in the non-severe asthmatic subtype compared to HCs. Interestingly, Spearman analysis revealed significant positive correlations among all tested HERV peptides within the Asthmatic cohort, a co-regulatory trend that became substantially reinforced within the Severe Asthma subgroup. These findings highlight a specific immune dysregulation pattern where a synchronized HERV expression tightly correlates with disease severity and phenotypical classification. The HERV epitopes identified in this exploratory study appear to be key targets of the humoral response in asthmatic patients, suggesting that circulating antibody levels against these retroviral elements could serve as potential tools for exploratory disease stratification and refractory phenotype characterization.

## Introduction

Bronchial asthma is a complex, heterogeneous chronic respiratory pathology characterized by variable and reversible airway obstruction, structurally underpinned by persistent mucosal inflammation [1]. This chronic inflammatory state drives bronchial hyperresponsiveness and drives long-term airway remodeling, which can progressively culminate in a fixed, irreversible loss of airflow dynamics [2]. The natural history of bronchial asthma is inherently punctuated by acute clinical exacerbations alternating with periods of total or partial symptom remission [3–5]. These acute episodes are triggered by a multitude of identifiable factors, spanning exogenous insults, such as airborne pollens, environmental pollutants, and viral respiratory infections, to endogenous shifts, including hormonal fluctuations, physical exertion, and gastroesophageal reflux disease (GERD), though a subset of cases remains cryptogenic [4, 6].

From an immunological perspective, asthma is currently stratified into two macroscopic pheno-endotypes: Type 2 (T2-high) and non-Type 2 (T2-low) inflammation, each governed by distinct upstream and downstream cellular networks [7, 8]. A universal histopathological hallmark in susceptible individuals is the prominent infiltration of the bronchial mucosa by an active cellular pool, primarily composed of recruited eosinophils, activated T lymphocytes, degranulated mast cells, and neutrophils [9, 10].

The development of severe, refractory asthma is heavily dictated by an intricate interplay between genetic predisposition and longitudinal environmental exposures [11–14]. Reflecting its complex polygenic etiology, genome-wide association studies (GWAS) have successfully identified over 200 genetic variants and approximately 128 independent loci linked to disease risk, confirming that hereditary susceptibility is not dictated by single monogenic drivers but resides within a dense, interconnected network of genomic interactions [4]. Crucially, this genetic architecture sharply differentiates childhood-onset from adult-onset asthma [15]. Childhood-onset asthma displays a three-fold higher genetic heritability and strong overlapping profiles with atopic comorbidities, largely driven by genes governing epithelial barrier integrity and allergic sensitization, such as the 17q21 locus (containing *ORMDL3* and *GSDMB*) and genes encoding alarmins or epithelial cytokines like Interleukin-33 (IL-33) and Thymic Stromal Lymphopoietin (TSLP) [9, 10, 16, 17]. Conversely, adult-onset asthma is strongly influenced by cumulative environmental factors and exhibits a distinct genomic framework focused on intrinsic parenchymal and localized pulmonary mechanics [4, 13].

The integration of functional epigenomic datasets has bridged the gap between environmental insults and altered gene translation [18, 19]. Epidemiological studies have consistently shown that the rising prevalence of allergic diseases is primarily driven by changing environmental dynamics, particularly a reduced exposure to protective microbial factors (e.g., traditional farming environments) alongside an increased exposure to modern risk factors such as urban pollution. These evolving environmental influences interact directly with individual genetic susceptibility through epigenetic mechanisms, positioning allergic disorders as a classic prototype of complex gene-environment interactions [19, 20]. Post-translational histone modifications, micro-RNA dynamics, and DNA methylation patterns act as critical biological mediators translating early-life environmental exposures (e.g., maternal smoking, urban pollution, and microbiome shifts) into stable, altered profiles of gene expression [21, 22]. Rather than acting solely through a simple Th1/Th2 shift, these epigenetic alterations shape a far more complex immune network, regulating innate lymphoid cells (ILCs) and the integrity of structural barrier tissues such as the airway epithelium [14, 19, 23]. While certain methylation marks possess predictive value, emerging evidence indicates that a significant proportion of epigenetic variance is a direct consequence of the localized inflammatory cascade itself, highlighting the potential of these molecular modifications to serve as sensitive disease biomarkers [24, 25]. In addition, these mechanisms critically regulate the immune balance between Th1 and Th2 cell responses and influence key structural barriers such as epithelial cells. Since epigenetic modifications are inherently dynamic and reversible, they not only capture past environmental exposures or reflect active inflammation but also represent promising therapeutic targets for early-life preventive strategies (such as dietary or microbial interventions) and personalized treatments based on endotypes [19]. This is especially relevant in modern westernized environments, where climate-change mitigation measures and energy-efficient building designs have shifted exposures toward poorly ventilated indoor spaces. Early-life epigenetic remodeling triggered by indoor pollutants in these environments can dictate distinct disease phenotypes, ultimately extending the scope of the Hygiene Hypothesis to a broader spectrum of chronic inflammatory conditions [23]. Within this highly interconnected gene-environment landscape, attention has increasingly shifted toward the “dark genome,” specifically Human Endogenous Retroviruses (HERVs) [26, 27]. Accounting for approximately 8% of the total human genome, HERVs are hereditary remnants originating from ancient exogenous retroviral infections that integrated into the human germline millions of years ago [28]. Historically dismissed as non-functional evolutionary “viral fossils,” it is now firmly established that HERVs are transcriptionally active and intimately involved in host immunomodulation [29].

Aberrant HERV expression has been tightly linked to the pathophysiology of several severe autoimmune, neurodegenerative, and chronic inflammatory diseases [30]. For instance, the envelope (Env) protein of HERV-W has been identified as a potent pro-inflammatory driver in Multiple Sclerosis (MS) and Type 1 Diabetes, where it acts as an endogenous agonist capable of triggering Toll-like Receptor 4 (TLR4) on innate immune cells, thereby inducing a potent cascade of pro-inflammatory cytokines [31–33]. Similarly, HERV-K activation has been characterized in the pathogenesis of Systemic Lupus Erythematosus (SLE) and Rheumatoid Arthritis, where retroviral antigens contribute to molecular mimicry phenomena, breaking immunological tolerance and promoting the synthesis of cross-reactive autoantibodies [34–36]. In the context of neurodegeneration, aberrant HERV-K activation has been documented to disrupt neuronal integrity, particularly in Amyotrophic Lateral Sclerosis (ALS), where envelope protein expression drives neurotoxicity and neuroinflammation [37, 38]. Furthermore, aberrant activation of HERV-H elements has been increasingly recognized in chronic inflammatory oncogenic microenvironments; acting primarily through non-coding RNAs, HERV-H expression alters cellular differentiation pathways and promotes immune evasion, further highlighting the diverse mechanistic repertoires of retroviral reactivations in human disease [39–41].

Because HERVs are strictly controlled by epigenetic silencing mechanisms, such as DNA methyltransferases (DNMTs) and histone deacetylases (HDACs), any sustained disruption in the host epigenetic machinery can prompt their transcriptional reactivation [42]. In chronic inflammatory conditions, localized oxidative stress and dominant cytokine networks can alter chromatin accessibility, leading to the localized transcription of HERV elements [43].

To properly conceptualize these dynamics, the paradigm of the “exposome” has emerged as a cornerstone of modern respiratory medicine, capturing the cumulative lifespan environmental exposures an individual encounters [44]. In asthma research, the exposomic framework recognizes that disease severity and phenotype progression result from synergistic high-risk clusters, such as the overlapping impacts of traffic-related air pollution, tobacco smoke, and recurrent viral infections [45, 46]. This framework offers a robust theoretical structure for analyzing how environmental insults modulate genetic susceptibility during critical windows of vulnerability.

Crucially, analyzing internal biomarkers like HERVs within this exposomic structure offers a direct window into how external environmental pressures translate into specific internal molecular signatures and disease phenotypes. The complexity of asthma pheno-endotyping continues to raise questions regarding the upstream biological mechanisms driving disease progression, particularly concerning the activation of divergent inflammatory pathways and refractory phenotypes [47–49]. While literature investigating this retroviral-exposome connection in asthma remains in its infancy, pivotal recent evidence by Reddam et al. demonstrated that indoor air pollution could significantly remodel the DNA methylation profiles of HERV and LINE-1 elements in asthmatic children, directly impacting airway inflammation [27].

However, while previous studies have primarily evaluated the HERV methylome at a genomic DNA level, the downstream translation of these elements and the subsequent systemic humoral response remain completely uncharacterized in bronchial asthma [27]. To address this critical gap in translational medicine, this exploratory study aimed to evaluate for the first time the circulating humoral response against specific antigenic envelope peptides derived from three distinct retroviral families, HERV-H, HERV-K, and HERV-W, across different clinical asthma phenotypes, with a specific focus on severe refractory patients, utilizing healthy individuals as controls.

## Materials and methods

### Samples collection

We evaluated a group of 46 patients with non-severe asthma (31 females and 15 males; mean age ± SD: 51.26 ± 14.97 years), a group of 23 patients with severe asthma (13 females and 10 males; mean age ± SD: 60.59 ± 10.95) and a HCs group, matched for age and sex, recruited from the pulmonology clinic and the transfusion center of the AOU of Sassari (39 females and 26 males; mean age ± SD: 53.38 ± 13.255 years). The data are summarized in the Table 1.

**Table 1:** Demographic data of investigated groups, including gender distribution and age (Mean ± SD)

Study population	Male	Female	Mean Age ± SD
Asthma	15	31	51.26 ± 14.97
Severe Asthma	10	13	60.59 ± 10.95
Healthy controls	26	39	53.38 ± 13.25

The population in question was sampled between September 2025 and October 2025, and included patients with non-severe asthma and severe asthma diagnosed and examined at the Pulmonology Clinic of the University Hospital of Sassari. The diagnosis and severity of asthma were assessed in accordance with the GINA, ATS, and ERS guidelines, based on clinical history, spirometry with bronchodilator reversibility, and the ACT questionnaire. Severe asthma was defined as uncontrolled disease even after optimized therapy with high doses of ICS/LABA (GINA levels 4–5). Patient follow-up included regular clinical evaluations, assessment of annual exacerbations, pulmonary function tests (FEV₁, FVC, PEF), and laboratory tests (total IgE and serum eosinophils).

Peripheral venous blood samples were collected in tubes containing K2-EDTA.

Whole blood was gently layered onto an equal volume of Ficoll (Sigma-Aldrich, St. Louis, MO, USA) in 15 mL tubes and centrifuged for 20 min at 1800 RPM without brake. The plasma, located in the upper layer, was removed by pipetting, and analyzed for the presence of antibodies against epitopes derived from the envelope of HERV-K, HERV-H, and HERV-W.

All subjects provided informed consent before participating in the study.

### Peptides

The peptides targeting analogous regions of the envelope (Env) protein—specifically HERV-K (19–37), HERV-H (229–241), and HERV-W (248–262) were designed using the Immune Epitope Database (IEDB) database and analysis tool and synthesized with > 95% purity (LifeTein, South Plainfield, NJ 07080, USA).

The IEDB software predicts the regions of proteins that are most likely to be recognized as epitopes in the context of a B cell response. All peptides were dissolved in DMSO and stored at − 20 °C in single-use aliquots (10 mM). (Table 2).

**Table 2:** Epitopes identified in HERV-K, HERV-H, HERV-W

	Amino Acid Sequence	Epitope Position	UniProtKb Accession Number
HERV-K	VWVPGPTDDRCPAKPEEEG	19–37	O42043
HERV-H	LGRHLPCISLHPW	229–241	Q9N2J8
HERV-W	NSQCIRWVTPPTQIV	248–262	Q9UQF0

### Enzyme-linked immunosorbent assay

The indirect enzyme-linked immunosorbent assay (ELISA) was used to quantify specific IgG antibodies directed against the HERV-K (19 37), HERV H (229 241), and HERV W (248 262) epitopes. First, 96-well Nunc plates were coated with a carbonate bicarbonate buffer solution (0.05 M, pH 9.5) containing the respective peptides at a concentration of 10 µg/mL; overnight incubation at 4 °C is necessary to allow optimal adhesion of the antigens to the plastic surface. Subsequently, to saturate non-specific binding sites, each well was incubated for one hour at 37 °C with a blocking solution consisting of 1% skimmed milk powder in 1× TBS (Tris-buffered saline). This step was essential to saturate the free sites not occupied by the peptide, reducing the assay background. Next, the plasma samples, diluted 1:100 in the same blocking solution, were transferred to the wells and incubated for one hour at 37 °C to promote the binding of the antibodies present in the sample to the immobilized epitopes. After the incubation phase with the sample, four washing cycles were performed using TBS containing 0.05% Tween 20 (TBS T) to remove unbound antibodies and minimize non-specific binding. Next, a secondary anti-human IgG antibody (Sigma-Aldrich, St. Louis, MO, USA; Cat. No. A1543), conjugated with alkaline phosphatase (1:1000 dilution) was added to a solution containing 1% milk in 1× TBS; the wells were incubated for 30 min at 37 °C. After further washing with TBS T, the substrate p-nitrophenyl phosphate (Sigma-Aldrich p-Nitrophenyl Phosphate Liquid Substrate System N7653-100ML- pNPP) dissolved in Milli Q water was added and the reaction was allowed to develop in the dark for 10 min. The reaction was stopped with 3 N NaOH. The optical density was measured at 405 nm using a microplate reader (Agilent BioTek 800 TS). Each sample was analyzed in duplicate; normalization was performed using a positive control included in each plate, while the background was defined as the average value obtained from wells containing only the secondary antibody on immobilized peptide (without primary antibody). The results are expressed as the average of the OD (Optical Density) values at 405 nm of the duplicates, after subtraction of the background.

### Statistical analysis

Statistical analysis was performed using GraphPad Prism 10 (GraphPad Software, San Diego, CA, USA). Given the data with non-normal distribution, comparisons between two independent groups (such as in the supplementary analyses) were evaluated using the Mann–Whitney U test, whereas multi-group comparisons among HCs, non-severe asthma, and severe asthma were evaluated using the Kruskal–Wallis test followed by Dunn’s multiple comparisons test. Cut-off values for categorical analyses were established based on Receiver Operating Characteristic (ROC) curve analysis, selecting the threshold that yielded a sensitivity greater than 90%, and differences in proportions were evaluated using Fisher’s exact test. A Spearman correlation test was performed among levels of antibodies to HERV-K, HERV-W, and HERV-H. Statistical significance was set as a p-value < 0.05.

## Results

We enrolled 46 patients with non-severe asthma, 23 with severe asthma (Table 3), and 65 matched HCs.

**Table 3:** Asthma and severe asthma patients’ clinical data

ID Patients	Gender	Y/o	Early onset Asthma	Allergic condition	Asthma (type)	Severe Asthma	Inhaled Therapy
A01	M	63	0	1	type 2	0	ICS/LABA
A02	F	46	1	1	type 2	0	ICS/LABA
A03	F	76	0	1	type 2	0	ICS/LABA
A04	M	48	1	1	type 2	0	ICS/LABA
A05	F	44	0	1	type 2	0	ICS/LABA
A06	F	28	1	1	type 2	0	ICS/LABA
A07	F	29	1	1	Type 2/ non type 2	0	ICS/LABA
A08	M	34	1	1	type 2	0	ICS/LABA
A09	F	77	1	1	type 2	0	ICS/LABA
A10	M	64	0	1	type 2	0	ICS/LABA/LAMA
A11	M	72	0	1	type 2	0	ICS/LABA
A12	M	56	0	0	non type 2	0	ICS/LABA
A13	F	48	0	1	type 2	0	ICS/LABA
A14	F	33	1	1	type 2	0	ICS/LABA
A15	M	53	0	1	type 2	0	ICS/LABA
A16	F	47	1	1	type 2	0	ICS/LABA
A17	M	38	1	1	type 2	0	ICS/LABA
A18	F	31	0	1	type 2	0	ICS/LABA
A19	M	47	1	1	type 2	0	
A20	F	65	0	1	type 2	0	ICS/LABA
A21	F	60	1	1	type 2	0	
A22	M	68	0	1	type 2	0	ICS/LABA
A23	F	39	1	1	type 2	0	ICS/LABA
A24	F	43	0	1	type 2	0	ICS/LABA
A25	M	37	1	1	type 2	0	SABA
A26	M	76	0	1	type 2	0	ICS/LABA
A27	F	57	0	1	type 2	0	ICS/LABA
A28	F	35	0	1	type 2	0	SABA
A29	M	42	1	1	type 2	0	ICS/LABA
A30	M	29	1	1	type 2	0	ICS/LABA
A31	F	53	1	1	type 2	0	
A32	F	35	1	1	type 2	0	SABA
A33	M	63	1	1	type 2	0	ICS/LABA
A34	F	50	0	1	type 2	0	ICS/LABA
A35	F	61	1	1	type 2	0	ICS/LABA
A36	F	58	1	1	type 2	0	ICS/LABA
A37	F	38	0	1	type 2	0	ICS/LABA
A38	F	77	0	0	non type 2	0	ICS/LABA
A39	F	54	0	0	non type 2	0	ICS/LABA/LAMA
A40	F	43	0	0	non type 2	0	ICS/LABA
A41	F	52	1	1	type 2	0	ICS/LABA/LAMA
A42	F	52	1	1	type 2	0	ICS/LABA
A43	F	27	1	1	type 2	0	ICS/LABA
A44	F	80	0	1	type 2	0	ICS/LABA/LAMA
A45	F	56	0	1	type 2	0	ICS/LABA
A46	F	69	0	1	type 2	0	ICS/LABA
AG01	F	74	0	1	type 2	1	ICS/LABA/LAMA
AG02	M	67	0	0	non type 2	1	ICS/LABA/LAMA
AG03	F	67	0	1	type 2	1	ICS/LABA/LAMA
AG04	F	66	0	1	type 2	1	
AG05	M	59	0	1	type 2	1	ICS/LABA
AG06	F	70	0	1	type 2	1	ICS/LABA
AG07	F	46	0	1	type 2	1	ICS/LABA/LAMA
AG08	M	65	0	0	non type 2	1	ICS/LABA/LAMA
AG09	F	55	0	1	type 2	1	ICS/LABA/LAMA
AG10	M	71	1	1	type 2	1	ICS/LABA
AG11	F	52	0	1	type 2	1	ICS/LABA/LAMA
AG12	F	81	0	1	type 2	1	ICS/LABA/LAMA
AG13	M	57	0	1	type 2	1	ICS/LABA
AG14	F	56	1	1	type 2	1	ICS/LABA/LAMA
AG15	F	69	1	1	type 2	1	ICS/LABA/LAMA
AG16	M	53	0	0	non type 2	1	ICS/LABA/LAMA
AG17	F	66	0	1	type 2	1	ICS/LABA/LAMA
AG18	M	67	0	1	type 2	1	ICS/LABA/LAMA
AG19	M	71	0	1	type 2	1	ICS/LABA/LAMA
AG20	F	31	1	1	type 2	1	0
AG21	F	51	0	0	non type 2	1	ICS/LABA/LAMA
AG22	M	62	1	1	type 2	1	ICS/LABA
AG23	M	51	0	1	type 2	1	ICS/LABA/LAMA

**Fig. 1:**
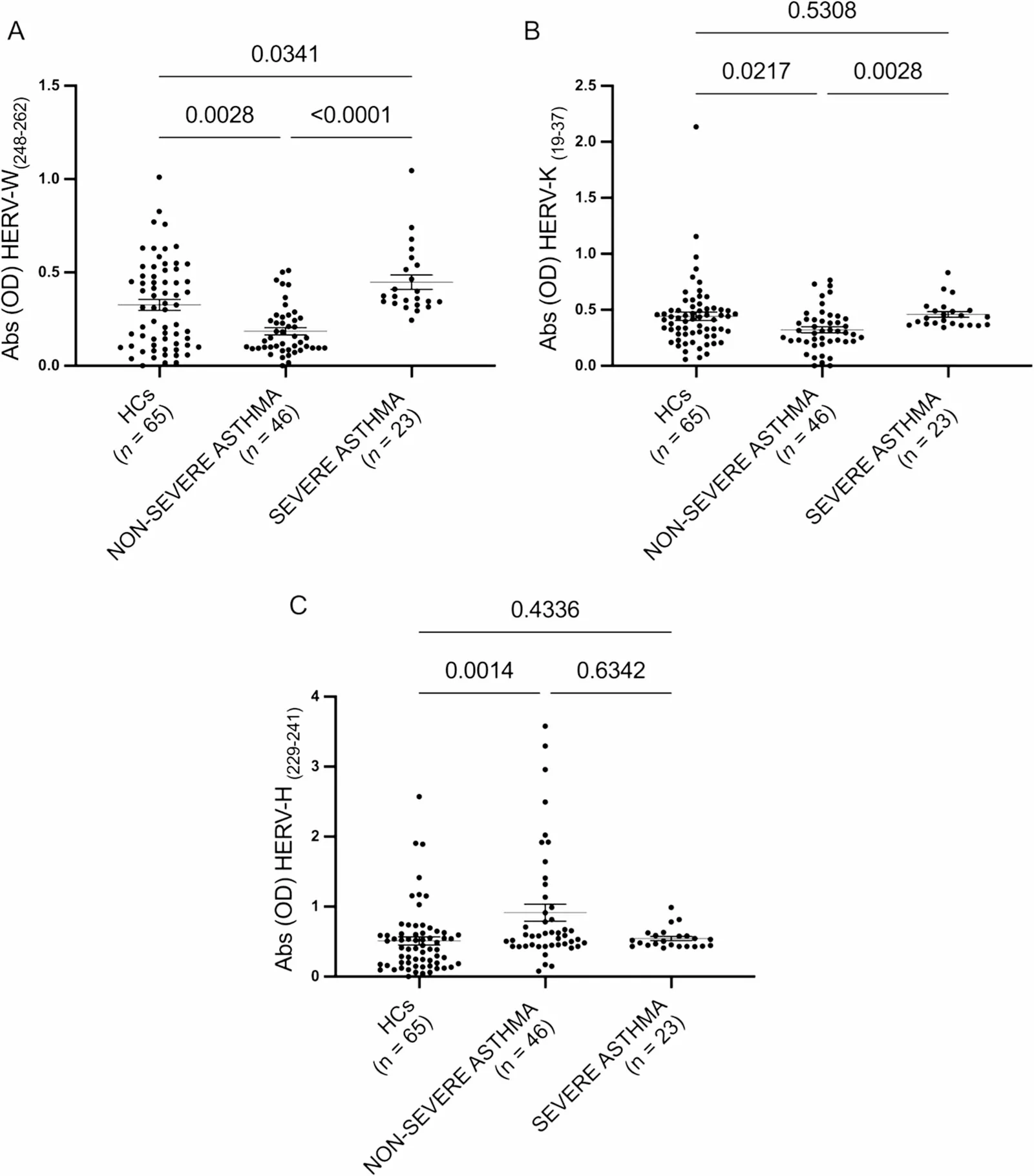
ELISA based analysis of Abs HERV-W, HERV-K and HERV-H. Serum antibody levels (expressed as optical density, OD) directed against envelope peptides derived from human endogenous retroviruses: HERV-W (248–262) (top left), HERV-K (19–37) (top right), and HERV-H (229–241) (bottom) in HCs (*n* = 65), patients with non-severe (mild-to-moderate) asthma (*n* = 46), and patients with severe asthma (*n* = 23). Horizontal lines represent mean with SE. Statistical significance between groups was determined using the Kruskal–Wallis test followed by Dunn’s multiple comparisons test, with significant p-values indicated above the horizontal comparison bars

Serum IgG responses against envelope peptides derived from HERV-W, HERV-K, and HERV-Hwere evaluated between HC (*n* = 65), patients with non-severe asthma (*n* = 46), and patients with severe asthma (*n* = 23). The Kruskal-Wallis test was used to assess statistical differences between groups, followed by Dunn’s multiple comparison test. Regarding HERV-W (248–262) (Fig. 1A), antibody levels showed a statistically significant difference between the study groups. Specifically, levels were significantly lower in the non-severe asthma group compared to HCs (*p* = 0.0028). In contrast, patients with severe asthma showed a marked increase in HERV-W antibody titers compared to the non-severe group (*p* < 0.0001), reaching values significantly higher than those observed in HCs (*p* = 0.0341). Similarly, analysis of HERV-K reactivity (19–37) (Fig. 1B) revealed a significant reduction in patients with non-severe asthma compared to HCs (*p* = 0.0217). In the severe asthma group, HERV-K antibody levels increased significantly compared to the non-severe subgroup (*p* = 0.0028), showing no statistically significant differences when directly compared with HCs (*p* = 0.5308).Finally, the evaluation of HERV-H (229–241) (Fig. 1C) showed a wider dispersion of values in the group with non-severe asthma, with no statistically significant differences observed between HC and non-severe asthma (*p* = 0.3510), or between HC and patients with severe asthma (*p* = 0.4536). However, a direct and statistically significant difference emerged when comparing the non-severe and severe asthma groups (*p* = 0.0014), highlighting a specific upregulation associated with disease severity phenotypes.

**Fig. 2:**
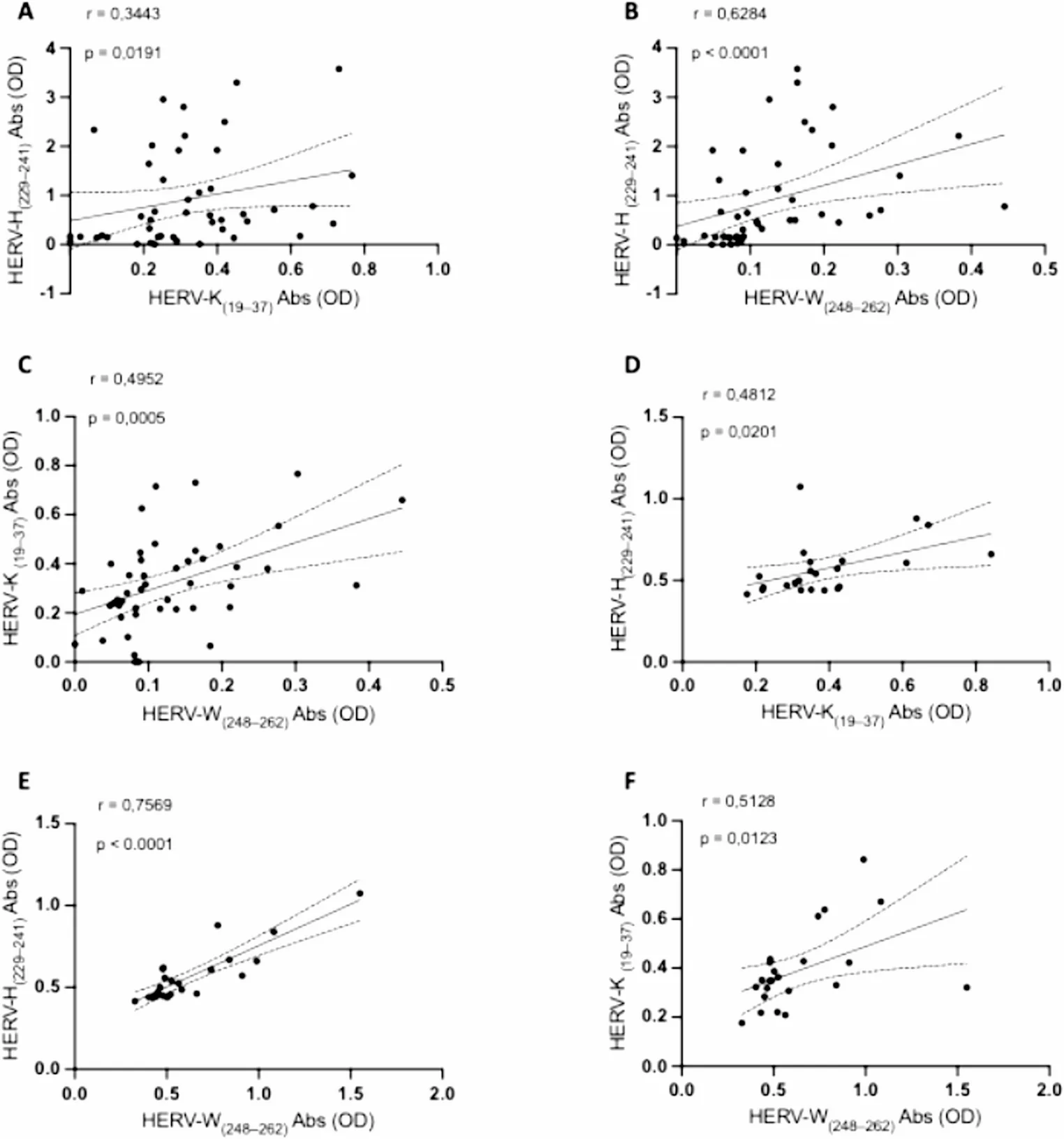
Spearman correlation analysis and co-regulatory dynamics among circulating anti-HERV antibodies.Linear regression models illustrating the mutual relationships between specific retroviral envelope-derived peptides within different clinical cohorts. **A–C** Inter-peptide correlation analysis in the Asthmatic patient group (*n* = 46), demonstrating significant positive associations between (**A**) anti-HERV-K and anti-HERV-H, (**B**) anti-HERV-W and anti-HERV-H, and (**C**) anti-HERV-W and anti-HERV-K antibody levels. **D–F** Inter-peptide correlation analysis within the Severe Asthma subgroup (*n* = 23), revealing a strongly reinforced co-regulatory network between (**D**) anti-HERV-K and anti-HERV-H, (**E**) anti-HERV-W and anti-HERV-H, and (**F**) anti-HERV-W and anti-HERV-K antibody titers. Individual values are expressed as Optical Density (OD). The solid lines represent the linear regression, and the dashed lines indicate the 95% confidence intervals. Spearman correlation coefficients (r) and statistical significance (p) are explicitly reported within each panel

To further investigate the relationship and potential synchronization between the immune responses directed against the different HERV antigens, a Spearman correlation analysis was performed (Fig. 2).

In the Asthmatic patient population (*n* = 46), significant positive correlations were confirmed among all three tested retroviral targets, indicating a shared physiological regulatory mechanism. Specifically, a significant positive correlation was observed between circulating anti-HERV-K and anti-HERV-H antibody levels (*r* = 0.3443, *p* = 0.0191; Fig. 2A). This coordinated trend was markedly stronger between anti-HERV-W and anti-HERV-H titers (*r* = 0.6284, *p* < 0.0001; Fig. 2B). Additionally, a highly significant positive association was validated between anti-HERV-W and anti-HERV-K antibody responses (*r* = 0.4952, *p* = 0.0005; Fig. 2C).

In the Severe Asthma subgroup (*n* = 23), this synchronized trend was substantially reinforced, with all correlation coefficients (r) uniformly increasing, suggesting that enhanced disease severity acts as a robust catalyst for a coordinated immune response. Within this severe cohort, a significant and strengthened correlation was established between anti-HERV-K and anti-HERV-H antibodies (*r* = 0.4812, *p* = 0.0201; Fig. 2D). Remarkably, the tightest and most robust coordinated humoral response was found between anti-HERV-W and anti-HERV-H titers, reaching an exceptionally high degree of co-regulation (*r* = 0.7569, *p* < 0.0001; Fig. 2E). Finally, a strong, statistically significant positive correlation was maintained between anti-HERV-W and anti-HERV-K responses (*r* = 0.5128, *p* = 0.0123; Fig. 2F).

## Discussions

The involvement of Human Endogenous Retroviruses (HERVs) in the pathogenesis of chronic inflammatory and autoimmune diseases has gained significant attention in recent years [50–52]. However, their specific role in the heterogeneous context of bronchial asthma remained largely unexplored. In this study, we characterized the circulating humoral immune response against envelope-derived peptides of HERV-W, HERV-K, and HERV-H in patients with different asthma phenotypes, revealing distinct, expression-specific patterns that correlate tightly with clinical severity.

An important finding of our study is the distinct behavior of antibody titers in different asthma phenotypes. For HERV-W (248–262), antibody levels were significantly lower in the non-severe asthma group than in HCs but showed a marked and progressive increase in patients with severe asthma, reaching significantly higher values than both the non-severe subgroup and the HCs. Although HERV-W envelope expression has traditionally been linked to proinflammatory cascades, superantigen-like properties, and Toll-like receptor 4 (TLR4) activation in neuroinflammatory and autoimmune disorders such as multiple sclerosis and type 1 diabetes, its dual modulation in asthma represents an interesting scenario [53, 54]. This biphasic pattern, characterized by initial downregulation at mild to moderate stages that may reflect localized tissue sequestration of antigen within the bronchial mucosa or early immune tolerance phenomena, followed by significant intensification and upregulation observed in severe asthma, indicates a disruption of immune surveillance or a secondary amplification cycle driven by chronic treatment-refractory airway damage and persistent oxidative stress [54, 55].

Similarly, anti-HERV-K (19–37) antibody levels were significantly lower in the non-severe asthma group compared to HCs. Because HERV-K is the most evolutionarily recent and biologically active retroviral family in the human genome, a drop in physiological antibody titers might indicate an underlying state of immune dysregulation or an impaired surveillance mechanism in asthmatic individuals, limiting their capacity to maintain physiological reactivity against these ancient viral elements [28, 56]. In the severe asthma group, HERV-K antibody levels increased significantly compared to the non-severe subgroup, mirroring the trend observed for HERV-W and suggesting a generalized breakthrough of retroviral expression as disease severity escalates.

Finally, evaluation of HERV-H (229–241) showed no statistically significant differences when comparing HCs to non-severe or severe asthma groups individually, but a direct, statistically significant difference emerged when comparing non-severe and severe asthma groups directly. HERV-H elements are known to be tightly linked to active immune modulation, cellular reprogramming, and pluripotency networks. In the context of severe asthma, the specific shift observed between non-severe and severe phenotypes could be driven by the distinct, steroid-resistant inflammatory microenvironment and advanced tissue remodeling characteristic of severe refractory disease [57].

Furthermore, given that our adult cohort was recruited in Sardinia, Italy, it is essential to contextualize these findings within the island’s unique genetic and epidemiological landscape. Sardinia represents a classic, textbook example of accelerated genetic drift in human populations. Due to its unique geography and prolonged historical isolation, the island has functioned as a genetic isolate, resulting in an atypical constellation of allele frequencies compared to mainland Europe [58]. Consequently, it is both epidemiologically plausible and genetically grounded that adult asthmatics in Sardinia may experience elevated rates of T2-high asthma or present with more pronounced T2-high symptoms driven by the island’s distinct genetic architecture. While adult-onset asthma globally is frequently associated with T2-low mechanisms (such as neutrophilic or obesity-related pathways) [14], Sardinia’s history of intense genetic drift and environmental selection alters this standard paradigm. Adult asthmatics in Sardinia may present with classic T2-high biomarkers at a higher frequency than their counterparts on the European mainland, including elevated peripheral blood eosinophil counts, increased fractional exhaled nitric oxide (FeNO) levels, and high rates of comorbid allergic rhinitis. This specific underlying genetic and immune profile might directly influence retroviral transactivation and host humoral responses. Alternatively, this early suppression could also be modulated by long-term treatment with inhaled or systemic corticosteroids (ICS) extensively utilized by our patient cohort to manage chronic inflammation, an aspect that warrants further investigation in untreated cohorts.

Beyond the individual alterations of each retroviral target, our study provides the first exploratory evidence of a highly synchronized humoral mechanism in asthmatic pathology. The Spearman correlation analysis revealed significant positive correlations among all three tested HERV peptides within the Asthmatic population, indicating a shared physiological regulatory mechanism. Specifically, significant positive coregulations were validated between anti-HERV-K and anti-HERV-H, anti-HERV-W and anti-HERV-K, and anti-HERV-W and anti-HERV-H. Intuitively, this systemic coregulation suggests that the transcriptional activation or subsequent antigen presentation of these retroviral envelope elements does not occur in isolation. Instead, they appear to be driven by shared, overarching pathophysiological triggers, such as systemic oxidative stress, epigenetic chromatin remodeling, or specific transcription factors (e.g., NF-κB and STAT pathways) activated during chronic airway inflammation [54].

Intriguingly, this synchronized trend becomes visibly stronger and highly consolidated within the Severe Asthma subgroup, where the correlation coefficients uniformly increase. Most notably, the association between anti-HERV-W and anti-HERV-H antibodies reaches an exceptionally high and robust level of co-regulation (*r* = 0.7569, *p* < 0.0001). This progressive reinforcement of the correlation network in severe cases indicates that enhanced disease severity acts as a powerful catalyst for a coordinated immune response [59, 60]. The strict preservation and dramatic enhancement of their mutual correlations demonstrate that the underlying epigenetic dysregulation governing HERV expression becomes highly consolidated and uniform in the most severe, refractory phenotypes of bronchial asthma. While these findings arise from a geographically localized and numerically limited severe cohort, the high statistical significance of these synchronized networks justifies further investigation in wider, multi-center longitudinal studies.

Several limitations of the present study should be acknowledged. First, its cross-sectional design based on a single time-point measurement precludes evaluating the dynamic evolution of antibody titers over time. Second, the relatively small cohort size limited our statistical power for detailed subgroup analyses, such as stratifying by specific treatment regimens or clinical phenotypes. Furthermore, because all enrolled asthma patients were undergoing active pharmacological therapy (including ICS/LABA and biologics), we cannot completely rule out the modulating effect of treatment on epigenetic silencing and HERV expression. Future prospective studies enrolling treatment-naïve, newly diagnosed patients with longitudinal follow-up will be essential to delineate the precise trajectory of HERV activation throughout the disease course.

## Conclusions

In conclusion, our study demonstrates that circulating antibody profiles against HERV-W, HERV-K, and HERV-H vary significantly according to asthma severity, exhibiting distinct expression patterns and a characteristic biphasic modulation. In particular, the specific upregulation of anti-HERV targets in severe asthma, paired with a significantly reinforced co-regulatory correlation network within the refractory phenotype, highlights these retroviral elements as potential exploratory non-invasive biomarkers for disease stratification. Further longitudinal studies on larger patient cohorts are warranted to elucidate the precise molecular mechanisms triggering HERV expression in the airways, to completely isolate the impact of different pharmacological treatments, and to evaluate whether these retroviral targets could guide personalized therapeutic interventions.

## Data Availability

No datasets were generated or analysed during the current study.
